# Supplementary figures and images for: Endocranial anatomy of the ceratopsid dinosaur Triceratops and interpretations of sensory and motor function
Source: PeerJ. 2020 Sep 18;8:e9888. doi: 10.7717/peerj.9888 (PMC7505063; doi:10.7717/peerj.9888)

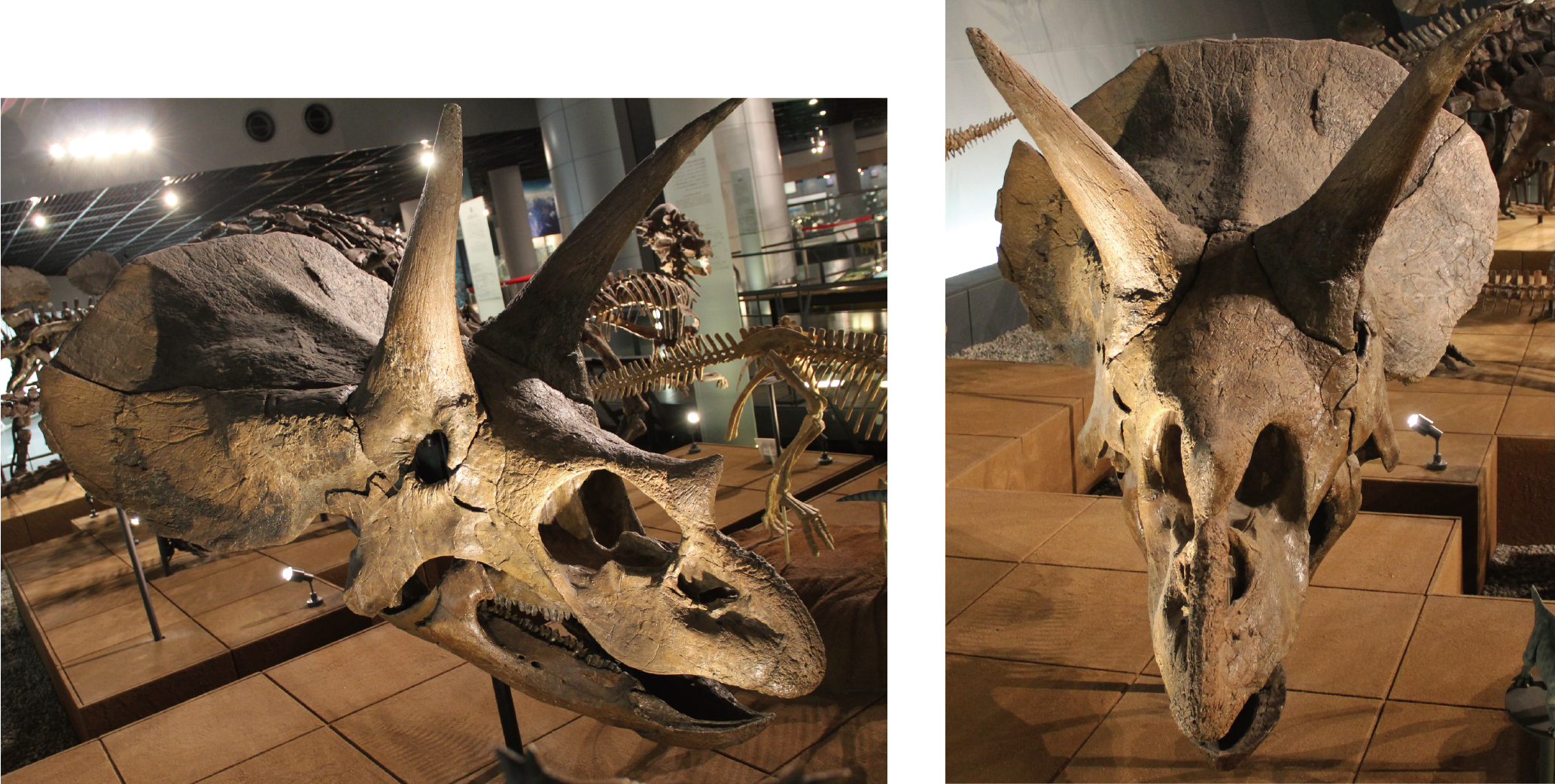

Supplement: Supplemental Information 1 [file peerj-08-9888-s001.png]

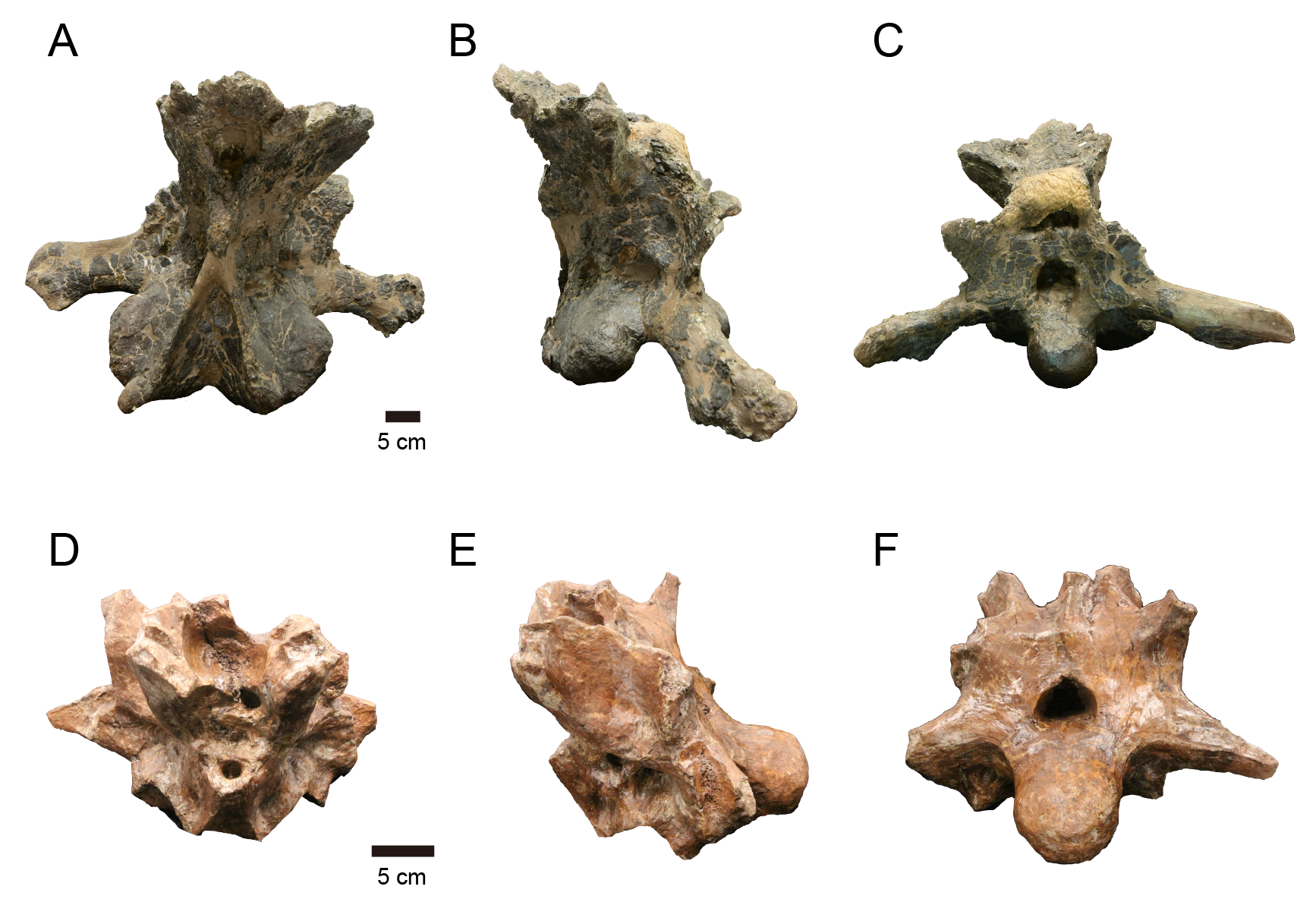

Supplement: Supplemental Information 2 — (A, D) anterior, (B, E) left lateral, (C, F) posterior views. [file peerj-08-9888-s002.png]
